# Supplementary material for: Predicting visual function by interpreting a neuronal wiring diagram
Source: Nature. 2024 Oct 2;634(8032):113–23. doi: 10.1038/s41586-024-07953-5 (PMC11446822; doi:10.1038/s41586-024-07953-5)
Supplement: Supplementary file 6 — CRF and ERF predictions for individual TmY4 and TmY9 cells. Analogous to Supplementary Data 3, but for TmY target types. Shown are the top four monosynaptic pathways, the strongest pathway passing through each of the top ten intermediary types (ranking from Extended Data Fig. 7), and the trisynaptic pathway Tm1–TmY–Dm3–TmY (see the section entitled Prediction of spatial normalization). [file 41586_2024_7953_MOESM6_ESM.zip › DataS4/TmY9q/720575940646898723.pdf]

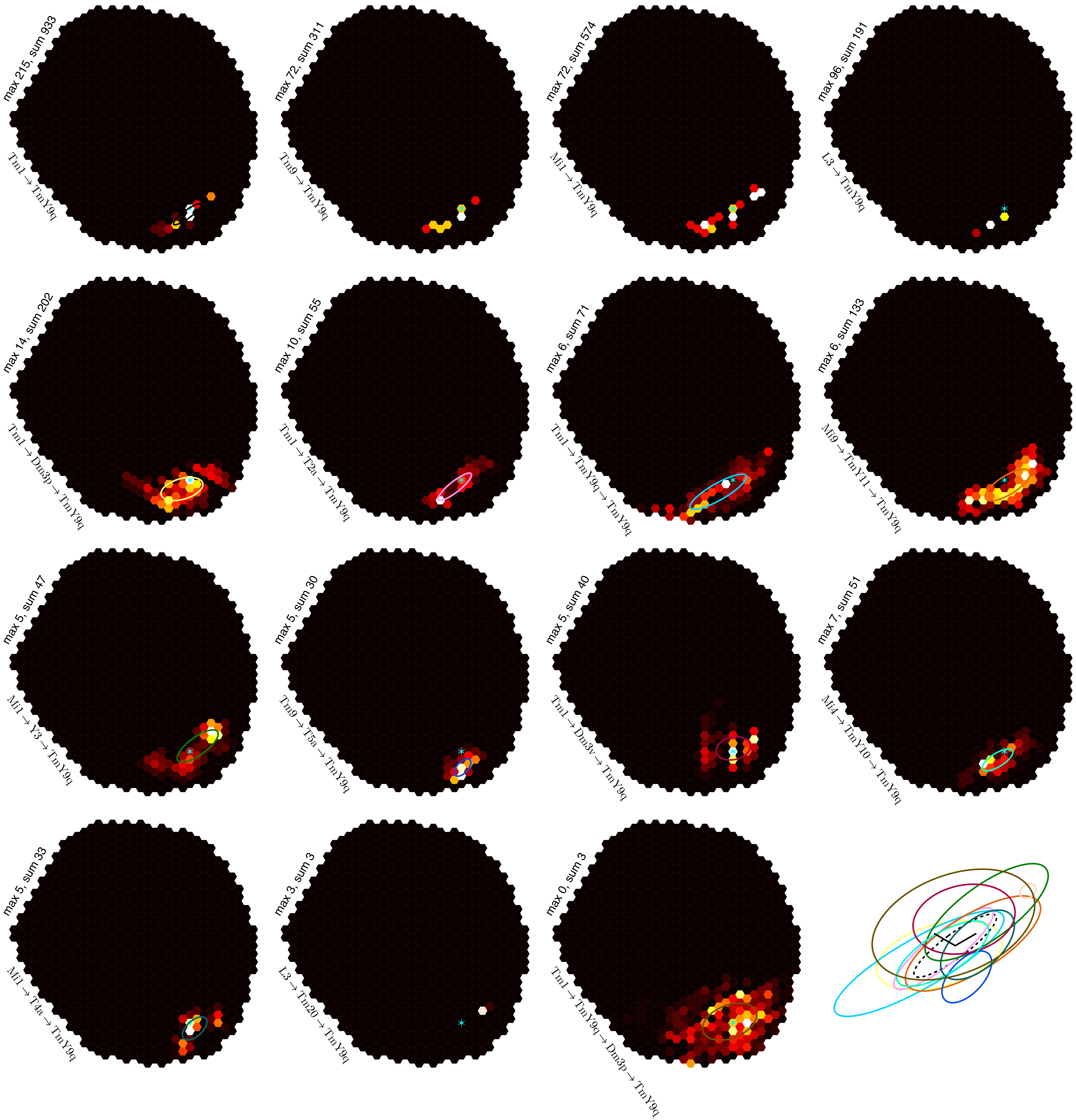
max 215, sum 933
Tm1 → TmY9q
max 72, sum 311
Tm9 → TmY9q
max 72, sum 574
Mi1 → TmY9q
max 96, sum 191
L3 → TmY9q
max 14, sum 202
Tm1 → Dm3p → TmY9q
max 10, sum 55
Tm1 → T2a → TmY9q
max 6, sum 71
Tm1 → TmY9q → TmY9q
max 6, sum 133
Mi9 → TmY11 → TmY9q
max 5, sum 47
Mi1 → Y3 → TmY9q
max 5, sum 30
Tm9 → T5a → TmY9q
max 5, sum 40
Tm1 → Dm3v → TmY9q
max 7, sum 51
Mi4 → TmY10 → TmY9q
max 5, sum 33
Mi1 → T4a → TmY9q
max 3, sum 3
L3 → Tm20 → TmY9q
max 0, sum 3
Tm1 → TmY9q → Dm3p → TmY9q
